# Immune-DDR crosstalk in pre-leukemia stem cells

**DOI:** 10.18632/oncotarget.21047

**Published:** 2017-09-19

**Authors:** Limei Wu, Allison Cole, Wei Du

**Affiliations:** *Wei Du*: Department of Pharmaceutical Sciences, School of Pharmacy, West Virginia University, Morgantown, WV, USA

**Keywords:** hematopoietic stem cell, leukemia stem cell, DNA damage response, immune response, pre-leukemia stem cells

## Hematopoietic stem cell and leukemia stem cell

Hematopoietic stem cells (HSCs) are rare, multipotent cells defined functionally by their ability to self-renew and differentiate into all mature blood lineages. Normal HSCs reside in a specialized bone marrow (BM) microenvironment (niche) that crucially regulates HSC survival and function. Many cell types including osteoblastic, perivascular, endothelial and mesenchymal cells contribute to the HSC niche. Signals derived from the HSC niche are necessary to regulate demand-adapted responses of HSCs and progenitor cells after BM stress or during infection.

Similar to normal hematopoiesis, leukemia is also hierarchically organized. A subpopulation of leukemic cells, leukemic stem cells (LSCs), is responsible for disease initiation and maintenance. Although LSCs harbor genetic abnormalities and give rise to more differentiated, heterogenic leukemic blasts, they share many characteristics with normal HSCs, including quiescence, multipotency and self-renewal. Strong evidence suggest that LSCs depend, at least in part, on similar signals from the microenvironment as HSCs do. However, they hijack the niche and the signaling molecules from normal HSCs during leukemogenesis.

## 

### DNA damage response in HSCs and LSCs

DNA damage repair (DDR) mechanisms are vital to maintain genomic integrity, which is of outmost importance for the survival at the cellular and organismal level and key to human health. The enormous regenerative potential coupled with lifetime persistence of HSCs may be particularly susceptible to DNA damage [1]. Maintenance of genomic integrity is a limiting factor in maintaining HSC function. It has also been postulated that HSCs, as compared to less primitive progenitors, exhibit delayed DNA double-strand break (DSB) rejoining, persistent DDR signaling activation, higher sensitivity to the cytotoxic effects of ionizing radiation, and attenuated expression of DNA-repair genes.

DNA damage accumulated in HSCs and progenitors is essential, at least in part, for the development of hematological malignancies. Ample evidence suggests that fundamental alterations in DNA surveillance and repair pathways in HSC, resulting in genetic instability, may ultimately lead to deregulated self-renewal, heralding the emergence of LSCs, which is the most significant event in the multistep pathogenesis of leukemogenesis [2, 3]. For example, a variety of inherited syndromes with DNA repair defects convey a high risk of myelodysplasia (MDS) and leukemia. Exposure to DNA-damaging agents, such as carcinogens and chemotherapeutics, increases the risk of acute myeloid leukemia (AML). In addition, a subset of AML cases, especially those that arise in the setting of exposure to DNA-damaging agents, exhibits complex clonal karyotypic abnormalities, indicating that DNA damage is not successfully repaired before the initiating LSCs divided.

## 

### Immune response in leukemia

Clinical and experimental studies have documented an intrinsic immune-mediated anti-leukemia response. For example, cytotoxic effector CD8^+^ T cells (CTLs) directed against leukemia antigens have been detected in chronic myeloid leukemia (CML) and AML patients. Depletion of CD8^+^ T cells by monoclonal antibody leads to rapid disease progression. Recently, several independent studies implicate a critical role of immune receptors, such as Toll-like receptors (TLRs), Leukocyte immunoglobulin like receptor 2 (LILRB2, mouse ortholog paired Ig-like receptor, PirB), T-cell immunoglobulin mucin receptor 3 (Tim-3) and B-lymphocyte-induced maturation protein 1 (Blimp-1), in leukemia development [4, 5].

While some leukemia antigens originate directly from the oncogenic event and are therefore leukemia-specific, the majority of leukemia-specific genetic alterations, such as chromosomal translocations, do not give rise to antigenic proteins. In addition, immune tolerance mechanisms that normally protect healthy tissues from autoimmune damage pose a challenging barrier to the development of effective anti-tumor immunity. Furthermore, emerging research has shown that leukemia cells engage in novel associations with reprogrammed stromal cells, including mesenchymal stromal/stem cells (MSCs), endothelia cells, T cells and myeloid cells that constitute the tumor microenvironment (TME), which is well-equipped to hinder the host immune response generated against tumor, and impede the efficacy of cancer immunotherapies.

## 

### The crosstalk between DDR and immune response

The crosstalk between DDR and immune response has been long appreciated. On one hand, exogenous or endogenous insults can stimulate DDR and trigger innate and adaptive immune responses to favor the immunogenicity of incipient cells. On the other hand, microbial infection is known to be sufficient to damage DNA in the nucleus and activate some or all of the DDR pathways. Disruption of DDR-immune response crosstalk compromises multicellular integrity and has been linked to cell cycle-related and immune defects.

Several independent studies have revealed the collaborative role of DDR and immune responses in leukemia development. Using a mouse model with long latency of mixed-lineage leukemia (MLL) development, Takavoval *et al.* identify DDR as a critical rate-limiting mechanism for malignant transformation by the MLL-Eleven nineteen leukemia (ENL) oncogene, synergizing with inflammatory factors in checkpoint signaling and senescence, thereby counteracting leukemogenesis [6]. Another study shows that Evi1-positive cells upregulated Bcl-2 and therefore, providing anti-apoptotic signals that amplifying the NRas-induced proliferative effect [7].

We recently investigated the relationship between DDR and leukemogenesis using the *Fanca* knockout mouse model, and show that the immune receptor Triggering receptor expressed on myeloid cells-1 (Trem1) cooperates with diminished DDR to induce pre-leukemic stem cell expansion [8]. The data raise an argument on the effectiveness of the cellular DDR machinery in preventing the transition of initiating pre-leukemic HSCs to a LSC population with transformed properties. Our findings implicate diminishing DDR as a root cause of leukemogenesis in genomic instability syndromes like Fanconi anemia, which subsequently cooperates with other signaling pathways for full transformation. The studies resemble the classical “two-hit” model of tumorigenesis, in which genetic deficiency leads to DDR loss followed by differentiation blockage and upregulation of proliferative signaling. We propose a model in which FA-deficient HSCs require both diminished DDR and enhanced immune response to become pre-leukemic. Therefore, targeting the collaborating signaling events in DDR-immune response crosstalk may open up a new avenue of therapeutic options for FA and other hematologic malignancies.
